# A Survey on Environmental Protective and Risk Factors and Awareness Related to Epithelial Barrier Integrity, Microbiome and Allergic Diseases

**DOI:** 10.1111/all.70190

**Published:** 2025-12-23

**Authors:** Arzu Bakirtas, Ayca Kiykim, A. Kubra Baskin, Hulya Anil, Hayrunnisa Bekis Bozkurt, Sevgi Sipahi Cimen, Zeynep Hizli Demirkale, Saliha Esenboga, Ismail Ogulur, Sena Ardicli, Deniz Cagdas, Walter Kistler, Hasan Yuksel, Cezmi A. Akdis

**Affiliations:** ^1^ Faculty of Medicine Department of Pediatric Allergy and Immunology Gazi University Ankara Türkiye; ^2^ Cerrahpasa School of Medicine, Pediatric Allergy and Immunology Istanbul University‐Cerrahpasa Istanbul Türkiye; ^3^ Pediatric Allergy and Immunology Osmangazi University Eskişehir Türkiye; ^4^ Division of Pediatric Allergy and Immunology, Department of Pediatrics Umraniye Training and Research Hospital Istanbul Türkiye; ^5^ Division of Pediatric Allergy and Immunology, Department of Pediatrics Sisli Etfal Training and Research Hospital Istanbul Türkiye; ^6^ Department of Immunology Istanbul University, Aziz Sancar Institute of Experimental Medicine Istanbul Türkiye; ^7^ Institute of Graduate Studies in Health Sciences Istanbul University Istanbul Türkiye; ^8^ Ihsan Doğramacı Children's Hospital, Department of Pediatrics, Division of Pediatric Immunology Hacettepe University Medical School Ankara Türkiye; ^9^ Department of Pediatric Basic Sciences, Division of Immunology Hacettepe University, Institute of Child Health Ankara Turkey; ^10^ Swiss Institute of Allergy and Asthma Research (SIAF), University of Zurich Davos Switzerland; ^11^ Department of Sports Medicine Davos Hospital Davos Switzerland; ^12^ Swiss Research Institute for Sports Medicine (SRISM) Davos Switzerland; ^13^ Medical Committee International Ice Hockey Federation (IIHF) Zurich Switzerland; ^14^ Medical Faculty, Department of Pediatric Allergy and Immunology Celal Bayar University Manisa Türkiye

**Keywords:** detergents, environmental pollutants, food processed, plastics, survey

To the Editor,

The epithelial barrier theory attributes the rise in allergic and other chronic diseases to environmental damage to epithelial surfaces and microbiota [1]. Common exposures—air pollutants, microplastics, detergents, and food additives—contribute to barrier dysfunction and microbial dysbiosis, linking them to allergic, autoimmune, and neuropsychiatric conditions [2].

Since the 1960s, allergic diseases such as asthma, rhinitis, atopic dermatitis, food allergy, and eosinophilic esophagitis have increased sharply, highlighting the environmental role beyond genetics [1, 3]. Factors like urbanization, processed foods, air pollution, and climate change worsen barrier integrity and microbial balance, fueling allergic inflammation [4, 5, 6].

The rising incidence and huge socioeconomic burden of up to 2 billion patients affected by these conditions highlight the urgent need for action. Global policy adjustments, stricter governmental regulations, and enhanced patient education are necessary to mitigate environmental risks, promote protective measures, and reduce allergic diseases. Identifying and modulating individual exposures to positive and negative factors, such as favorable lifestyle characteristics or certain toxic substances, as well as assessing these exposures, will increase awareness among patients and their families and represent a crucial step for future clinical trials and mechanistic studies. To address this need, two new questionnaires—one for parents and the other for adolescents—were developed by a multidisciplinary team. The authors are pediatric allergy and immunology specialists affiliated to the Experimental Allergy Working Group of the Society of Pediatric Allergy, Immunology and Asthma (CAIAD, a national society in Türkiye composed of pediatric allergy and immunology specialists) and international scientists with expertise in exposome and epithelial barriers. The group initially formed to investigate the role of the epithelial barrier in allergic diseases and identified a critical need to assess awareness of environmental and lifestyle factors potentially affecting barrier integrity. Since no validated tools existed, the team conducted a comprehensive literature review and translated relevant concepts into structured questionnaire items [1, 4, 5, 6]. The development process spanned approximately 10 months and included over a dozen structured meetings to ensure scientific accuracy and applicability.

The size and efficient applicability of the questionnaires were kept in focus during the preparation. Therefore, a multi‐step design was adopted. Both questionnaires start with demographic information and continue with two sections: Section 1. Exposures to environmental factors affecting epithelial barrier integrity and microbiome and Section 2. Awareness about the epithelial barrier theory. The first questionnaire is designed for parents, preferably mothers, of children and adolescents under 18 with allergic diseases (Supporting Information 1) (Figure 1). The second questionnaire is designed for adolescents and intended to be completed by themselves (Supporting Information 2) (Figure 2). The exposure questions in parents' questionnaire address items starting from the mother's pregnancy through the patient's current age including perinatal period, eating habits, and consumed food groups, cleaning and hygiene routines, use of plastic products, and exposure to air pollution. In the adolescent questionnaire, the exposure part included additional aspects of their current lifestyle habits that their parents may not be fully aware of such as personal care habits and physical activity questions. The personal care habits subsection covered the use of deodorants, perfumes, makeup, and hair dye products, as well as the presence of tattoos. The physical activity habits subsection explored the adolescent's regular engagement in exercise or sports activities, including frequency, duration, and type of activity. Also, the air pollution subsection was expanded beyond the parent survey to include questions about the adolescent's own use of tobacco‐related products. These questions assessed whether the adolescent smoked, the type of products used (e.g., cigarettes, e‐cigarettes, hookah), the amount consumed per day, and exposure to secondhand smoke.

**FIGURE 1:**
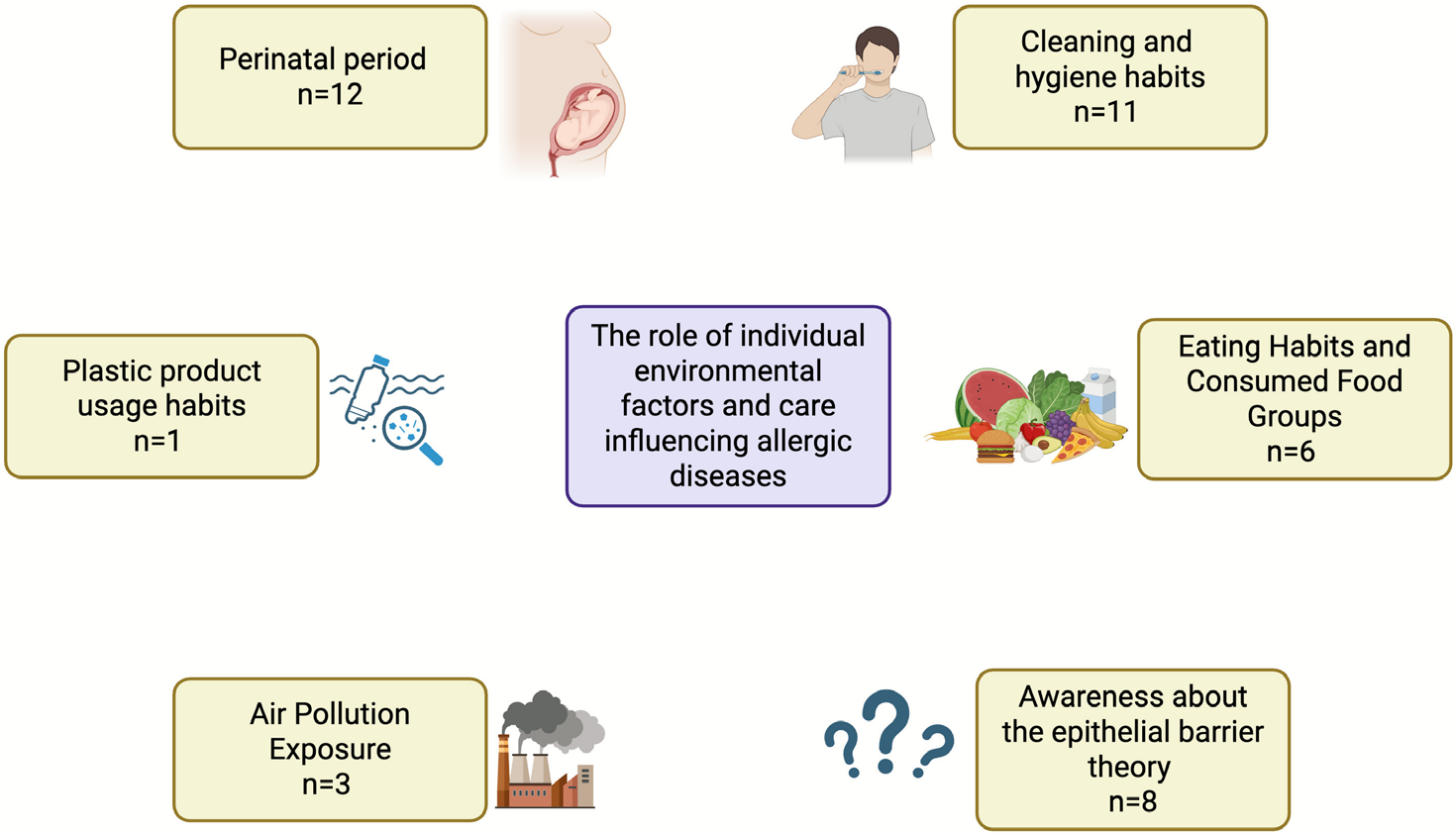
Structure of the parent‐completed questionnaire assessing awareness of the epithelial barrier theory and exposure to environmental factors from the perinatal period onward, including diet, hygiene practices, plastic use, and air pollution.

**FIGURE 2:**
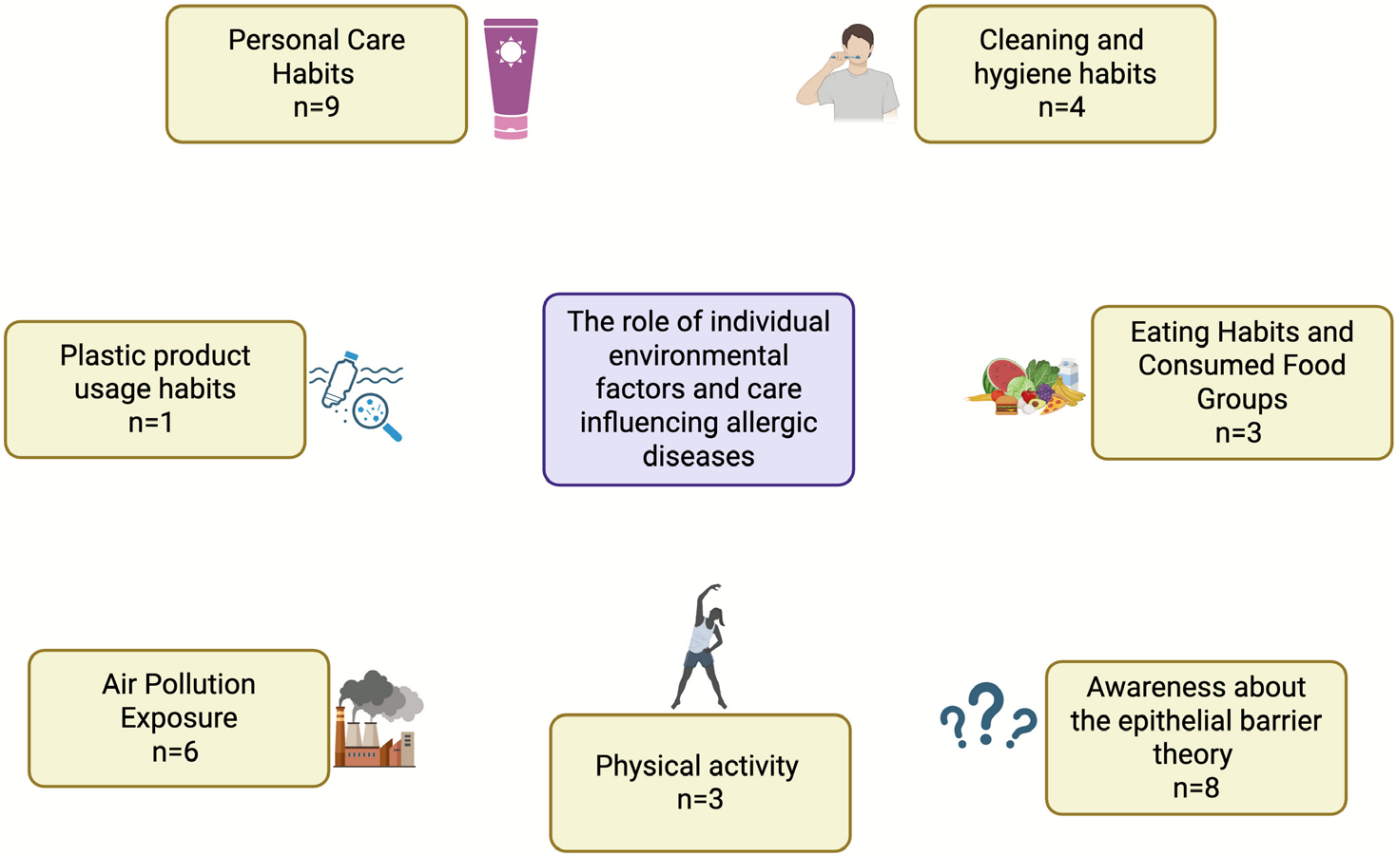
Structure of the adolescent‐completed questionnaire evaluating awareness of the epithelial barrier theory and current environmental exposures, including dietary habits, hygiene practices, plastic use, personal care habits, physical activity, and air pollution.

The awareness section begins with basic, universally understandable questions. Depending on responses, participants with prior knowledge or engagement are presented with follow‐up items exploring their information sources and behavior changes.

In order to enhance the purpose and applicability of the questionnaires, we developed a scoring system. The scoring system is applied only to the exposure section of both questionnaires. In this section, only the items with scientific evidence regarding their impact on epithelial barrier integrity and microbiome are included in the scoring. Accordingly, protective factors are assigned negative scores (−1 to −2), while risk factors are assigned positive scores (+1 to +2). The total score is then normalized to a scale ranging from 0 to 100, where higher scores indicate greater exposure to risk factors that may impair epithelial barrier integrity and microbiome (Supporting Information 3). The questionnaires are then field‐tested with 10 adolescents and 10 parents to assess the clarity of the items, language comprehensibility, and the approximate time required for completion. The next step will be to conduct formal validation and reliability studies following current best practices in scale development and validation [7, 8] which are planned as the immediate follow‐up project after this publication.

At this stage, physician‐reported items are excluded from the main versions to enhance broader applicability, but a supplementary clinician‐completed case report form for pediatric allergy patients has been provided as part of the study materials (Supporting Information 4).

As the authors, we declare that these two questionnaires are living documents that are open to the comments and suggestions from the readers and will continuously improve upon new ideas and scientific developments as environmental conditions and lifestyles change. While parent‐proxy and self‐reporting in adolescents may introduce recall bias, the findings will provide valuable insights to increase self‐awareness about the significance of the epithelial barrier integrity and microbiome. Importantly, these findings can encourage families and adolescents to take proactive steps to reduce their exposure to hazardous environmental exposure, contributing to developing improved policies to address these critical issues.

## Author Contributions

Conceptualization: Arzu Bakirtas and Cezmi A. Akdis. Data Curation: Arzu Bakirtas, Ayca Kiykim, A. Kubra Baskin, Hulya Anil, Hayrunnisa Bozkurt, Sevgi Sipahi Cimen, Zeynep Demirkale, Saliha Esenboga, Ismail Ogulur, Sena Ardicli, Deniz Cagdas, Walter Kistler, and Hasan Yuksel. Resources: Arzu Bakirtas, Ayca Kiykim, A. Kubra Baskin, Hulya Anil, Hayrunnisa Bozkurt, Sevgi Sipahi Cimen, Zeynep Demirkale, Saliha Esenboga, Ismail Ogulur, Sena Ardicli, Deniz Cagdas, Walter Kistler, and Hasan Yuksel. Supervision: Cezmi A. Akdis. Writing, Original Draft Preparation: Arzu Bakirtas and Cezmi A. Akdis.

## Funding

The authors received no specific finding for this work.

## Conflicts of Interest

Cezmi A. Akdis has received research grants from the Swiss National Science Foundation, European Union (EU CURE, EU Syn‐Air‐G), Novartis Research Institutes (Basel, Switzerland), Stanford University (Redwood City, Calif), Seed Health (Boston, USA), AO Research Institute (Davos, Switzerland), and SciBase (Stockholm, Sweden). He is the Co‐chair of the EAACI Guidelines on Environmental Science in Allergic Diseases and Asthma; serves on the Advisory Boards of Sanofi/Regeneron (Bern, Switzerland, New York, USA), Stanford University Sean Parker Asthma Allergy Center (CA, USA), Novartis (Basel, Switzerland), GlaxoSmithKline (Zurich, Switzerland), Bristol‐Myers Squibb (New York, USA), Seed Health (Boston, USA), and SciBase (Stockholm, Sweden). Cezmi A. Akdis is the Editor‐in‐Chief of Allergy. Arzu Bakirtas, Ayca Kiykim, A. Kubra Baskin, Hulya Anil, Hayrunnisa Bekis Bozkurt, Sevgi Sipahi Cimen, Zeynep Hizli Demirkale, Saliha Esenboga, Ismail Ogulur, Sena Ardicli, Deniz Cagdas, Walter Kistler, and Hasan Yuksel declare no relevant conflicts of interest.

## Supporting information


**Supporting Information: 1**. Survey for Parents.

**Supporting Information: 2**. Survey for Adolescents.

**Supporting Information: 3**. The Scoring system.

**Supporting Information: 4**. Case Report Form.

**Data S1:** Supporting Information.

**Data S2:** Supporting Information.

## Data Availability

Data sharing not applicable to this article as no datasets were generated or analysed during the current study.
