# Supplementary material for: A Survey on Environmental Protective and Risk Factors and Awareness Related to Epithelial Barrier Integrity, Microbiome and Allergic Diseases
Source: Allergy. 2025 Dec 23;81(3):930–3. doi: 10.1111/all.70190 (PMC12954555; doi:10.1111/all.70190)
Supplement: Supplementary file 3 — Supporting Information: 3. The Scoring system. [file ALL-81-930-s005.docx]

**Online Supplemental File 3**

**SCORING SYSTEM**

Below, we summarize the features and technical details of the new scoring system that are implemented in the manuscript:

**1. Missing responses:** Respondents are required to answer all questions; however, some items include an “I don’t know” or “I don’t remember” option. When these options are selected, the corresponding questions are excluded from the scoring.

.

**Normalization formula (0–100):** The 0–100 scale facilitates interpretation, reporting, and between group comparisons.

- For each respondent, we compute:

- raw_score = sum of item scores for answered items

- min_possible = sum of the minimum score for each answered item

- max_possible = sum of the maximum score for each answered item

- normalized_score = (raw_score − min_possible) / (max_possible −min_possible) × 100

**Example:** Scoring for "Parent respondent 1":

Perinatal period: -4

Eating Habits and Consumed Food Groups: -2

Cleaning and hygiene habits: 9

Plastic product usage habits: 1

Air pollution exposure: 2

Total raw score: 6

Min_possible score: -26

Max_possible score: 60

Normalized_score: (6 − (−26)) / (60 −(−26)) × 100 = 37.

**2.** No imputation is performed; exclusion prevents penalizing respondents for legitimately missing information. The total score is normalized based only on the questions a respondent actually answered, ensuring that selecting “I don’t know” and "I don't remember" items does not unfairly reduce their score.

**3.** We allowed each question to have its own scoring range, which now includes both positive and negative values. Directionality and polarity have been captured.

**4.** Dependent questions: Some questions are interrelated, where a response to one may influence the response to another. Each dependent question is scored separately to quantify the strength of the exposure.

**5.** Comparability and interpretability of the results are provided. By scaling each respondent’s total between the best and worst possible outcomes for their answered items, we produce consistent, comparable scores that reflect both performance and polarity, while maintaining fairness across incomplete responses.
