# Supplementary material for: A Survey on Environmental Protective and Risk Factors and Awareness Related to Epithelial Barrier Integrity, Microbiome and Allergic Diseases
Source: Allergy. 2025 Dec 23;81(3):930–3. doi: 10.1111/all.70190 (PMC12954555; doi:10.1111/all.70190)
Supplement: Supplementary file 4 — Supporting Information: 4. Case Report Form. [file ALL-81-930-s006.docx]

**CASE REPORT FORM**

**Study name:** A survey on environmental protective and risk factors and awareness related to epithelial barrier integrity, microbiome and allergic diseases

**Participant Code Number:**

**Age of the patient:**

**Gender of the patient:**

**ALLERGEN SENSITIZATIONS**

**Aeroallergen sensitization**

0: absent

1: present

**House dust mite** **sensitization**

0: absent

1: present

**Polen sensitization**

0: absent

1: present

**Pet sensitization**

0: absent

1: present

**Mold sensitization**

0: absent

1: present

**Food sensitization**

0: absent

1: present

**ASTHMA**

**Current doctor diagnosis of asthma and asthma control**

0: No doctor diagnosis of asthma

1: Doctor diagnosis of asthma, controlled

2: Doctor diagnosis of asthma, partially controlled

3: Doctor diagnosis of asthma, uncontrolled

4: Asthma in remission, off treatment for at least a year

**Number of asthma exacerbations requiring systemic corticosteroids in the last 12 months**

0: No exacerbation

1: One

2: Two

3: Three or more

**Current Status of Asthma Controller Therapy**

0: Not on asthma controller therapy

1: Receives Intermittent controller therapy (ICS / ICS formoterol or montelukast)

2: Receives Dailycontroller therapy (ICS / ICS formoterol or montelukast)

**Current ICS use for asthma**

0: No

1: Yes

**Current montelukast use for asthma**

0: No

1: Yes

**Current ICS / Formoterol use for asthma**

0: No

1: Yes

**Current ICS / salmeterol use for asthma?**

0: No

1: Yes

**Current biologic us efor asthma?**

0: No

1: Yes

**ALLERGIC RHINOCONJUNCTIVITIS (ARC)**

**Current doctor diagnosis of ARC and active symptoms**

0: No doctor diagnosis of allergic rhinoconjunctivitis or active symptoms

1: Doctor diagnosis of allergic rhinoconjunctivitis, but no active symptoms

2: Doctor diagnosis of allergic rhinoconjunctivitis, intermittent active symptoms

3: Doctor diagnosis of allergic rhinoconjunctivitis, persistent active symptoms

**Treatment of ARC**

0: No treatment

1: Intermittent treatment

2: Persistent treatment

**Oral antihistamines for ARC**

0: No

1: Yes

**Nasal corticosteroid for ARC**

0: No

1: Yes

**Nasal decongestants for ARC**

0: No

1: Yes

**Montelukast for ARC**

0: No

1: Yes

**Topical antihistamines for ARC (nasal or ocular)**

0: No

1: Yes

**Ocular corticosteroid for ARC**

0: No

1: Yes

**Allergen immunotherapy (AIT) for allergic rhinoconjunctivitis**

0: Never received AIT

1: Currently on AIT

2: Received AIT in the past

**ATOPIC DERMATITIS**

**Doctor diagnosis of atopic dermatitis and active symptoms**

0: No Doctor diagnosis of atopic dermatitis or active symptoms

1: Doctor diagnosis of atopic dermatitis but no active symptoms

2: Doctor diagnosis of atopic dermatitis and intermittent active symptoms

3: Doctor diagnosis of atopic dermatitis and persistent active symptoms

4: Atopic dermatitis is in remission, off treatment for at least a year

**Number of atopic dermatitis exacerbations requiring topical / systemic corticosteroid use**

0: No exacerbation

1: One

2: Two

3: Three or more

**Emolient Treatment Status for Atopic Dermatitis:**

0: Not receiving any treatment for atopic dermatitis

1: Receiving intermittent treatment for atopic dermatitis

2: Receiving continuous treatment for atopic dermatitis

**Treatment Status for Atopic Dermatitis (Excluding Emollients):**

0: Not receiving any treatment for atopic dermatitis

1: Receiving intermittent treatment for atopic dermatitis

2: Receiving continuous treatment for atopic dermatitis

**Proactive Treatment (with topical corticosteroids or topical calcineurin inhibitors) Status for Atopic Dermatitis:**

0: Not receiving proactive treatment

1: Currently receiving proactive treatment

2: Received proactive treatment in the past

**Cyclosporine Treatment Status for Atopic Dermatitis:**

0: Never received

1: Currently receiving

2: Received in the past

**Dupilumab Treatment Status for Atopic Dermatitis:**

0: Never received

1: Currently receiving

2: Received in the past

**IVIG Treatment Status for Atopic Dermatitis:**

0: Never received

1: Currently receiving

2: Received in the past

**FOOD ALLERGY**

**Doctor diagnosis of Food Allergy:**

0: Absent

1: Present

2: Tolerance developed

**Doctor diagnosis of Ig E mediated food allergy**

0: Absent

1: Present

2: Tolerance developed

**IgE mediated food induced urticaria/angioedema**

0: Absent

1: Present

2: Tolerance developed

**IgE mediated food induced anaphylaxis**

0: Absent

1: Present

2: Tolerance developed

**IgE mediated polen food allergy syndrome**

0: Absent

1: Present

2: Tolerance developed

**Non-IgE mediated food allergy**

0: Absent

1: Present

2: Tolerance developed

**Food protein induced allergic proctitis/procktocolitis**

0: Absent

1: Present

2: Tolerance developed

**Food protein induced enterocolitis syndrome (FPIES)**

0: Absent

1: Present

2: Tolerance developed

**Food protein induced enteropathy**

0: Absent

1: Present

2: Tolerance developed

**Mixed type food allergy**

0: Absent

1: Present

2: Tolerance developed

**Eosinophilic esophagitis (EoE)**

0: Absent

1: Present

2: Tolerance developed

**Eosinophilic gastrointestinal system diseases beyond EoE (other EGIS)**

0: Absent

1: Present

2: Tolerance developed

**Food triggered atopic dermatitis**

0: Absent

1: Present

2: Tolerance developed

**Current treatment for food allergy**

1: On one food elimination diet

2: On two food elimination diet

3: On three or more food elimination diet

**Oral allergen immune therapy (AIT) for food allergy**

0: No

1: Yes

**Current use of biologic for food allergy**

0: No

1: Yes

**DRUG ALLERGY**

**Doctor diagnosis of drug allergy**

0: No

1: Yes

2: Tolerance developed

**Early (Acute) onset drug allegy**

0: Absent

1: Present

2: Tolerance developed

**Drug induced anaphylaxis**

0: Absent

1: Present

2: Tolerance developed

**Drug induced urticaria and angioedema**

0: Absent

1: Present

2: Tolerance developed

**Late onset drug allergy**

0: Absent

1: Present

2: Tolerance developed

**Drug induced maculopapular eruption**

0: Absent

1: Present

2: Tolerance developed

**Drug induced severe cutaneous reactions (SJS, TEN, AGEP,DRESS, EM)**

0: Absent

1: Present

**Drug induced fixed eruptions**

0: Absent

1: Present

**Drug induced cytopenias**

0: Absent

1: Present

**Drug induced serum sickness disease and vasculitis**

0: Absent

1: Present

**Drug induced hepatitis, nephritis, interstisial lung disease**

0: Absent

1: Present

**Current treatment for drug allergy**

0: No treatment

1: One drug elimination

2: Two drug eliminations

3: Three and more drug eliminations

4: Under desensiization treatment

**CHRONIC URTICARIA**

**Doctor diagnosis of** **chronic spontaneous urticaria and active symptoms**

0: No chronic spontaneous urticaria or active symptoms

1: Chronic spontaneous urticaria but no active symptoms

2: Chronic spontaneous urticaria and active symptoms

3: Chronic spontaneous urticaria in remission off treatment for at least a year

**Current treatment status for chronic urticaria**

0: No treatment

1: Intermittent treatment

2: Persistent daily treatment

**Omalizumab treatment for chronic urticaria**

0: Never

1: On treatment

2: Received in the past

**Other systemic treatments for chronic urticaria**

0: Never

1: On treatment

2: Received in the past

**VENOM ALLERGY**

**Doctor diagnosis of Venom hypersensitivity**

0: No

1: Yes

**Urticaria and angioedema triggered by venom hypersensitivity**

0: No

1: Yes

**Venom induced anaphylaxis**

0: No

1: Yes

**Large local reaction with venoms**

0: No

1: Yes

**Venom allergen immunotherapiy (VIT)**

0: No

1: On VIT

2: Received VIT in the past

**HEREDITARY ANGIOEDEMA**

**Doctor diagnosis of Hereditary angioedema**

0: No

1: Yes

**Long term prophylactic treatment for Hereditary angioedema**

0: No

1: Yes

**MASTOCYTOSIS**

**Mastocytosis**

0: No

1: Yes

**Current treatment status for mastocytosis**

0: No

1: Yes
