# Supplementary material for: A Survey on Environmental Protective and Risk Factors and Awareness Related to Epithelial Barrier Integrity, Microbiome and Allergic Diseases
Source: Allergy. 2025 Dec 23;81(3):930–3. doi: 10.1111/all.70190 (PMC12954555; doi:10.1111/all.70190)
Supplement: Supplementary file 5 — Data S1: Supporting Information. [file ALL-81-930-s003.docx]

**Survey for Adolescents on Factors**

**Affecting the Epithelial Barrier Integrity, Microbiome and Allergic Diseases**

(This survey is designed for teens aged 12–18 years)

**Purpose of the Study:**

The inner surfaces of tissues such as the intestines, skin, and respiratory tract are lined with cells called epithelial cells. These cells form a barrier on the inner surfaces of the body, protecting us from microbes, toxins, and foreign substances.

The aim of our research is to assess the awareness of individuals in your age group regarding “epithelial cells (epithelial barrier), which form a boundary against microbes, toxins, and foreign substances, and their relationship with diseases,” as well as to evaluate your level of exposure to epithelial-disrupting substances.

Recent studies have shown that disruptions in this boundary maintained by epithelial cells may contribute to the development of many diseases. Through the questions in this survey and your responses, we aim to measure your level of knowledge on this topic and help raise awareness.

Participation in this study is entirely voluntary. The survey does not request any personal identifying information from you. The data you provide will not be linked to any personal information collected in the voluntary participation forms. Your responses will be kept strictly confidential and will only be evaluated by the researchers. The information obtained from this survey will be analyzed collectively and used in scientific publications.

I have read the above information and voluntarily agree to participate in this study.

Yes (Thank you for participating in our study)

No

**Participant code number:**

**General information**

1. Your age:………
2. Your sex:

- Male
- Female

1. Please select the option that best describes your level of education:

- Literate
- Primary school graduate
- Middle school
- High school

1. Do you live on a farm with livestock (e.g. cows, sheep, etc.) far from traffic?

- Yes
- No

1. How many people live in your household?

- 1–2
- 3–4
- 5–6
- 7 or more

1. Is there a grandfather or grandmother living with you in your home?

- Yes
- No
- Sometimes

1. How many siblings do you have?

- None
- One
- Two
- Three
- Four or more

1. How many of your siblings attend school/daycare?

- None
- One
- Two
- Three
- Four or more

1. Do your parents or siblings have any allergic diseases?

- Yes
- No (You can proceed to question 11)
- Not sure (You can proceed to question 11)

1. If yes, which allergic diseases are present? (You may select more than one option)

- Asthma
- Hay fever
- Chronic rhinosinusitis with or without nasal polyps
- Allergic eczema/atopic dermatitis
- Food allergy
- Drug allergy
- Bee allergy
- Urticaria (hives)
- Eosinophilic esophagitis
- Other

1. **Do any of the following diseases exist among immediate family members? (You may select more than one option)**

- High blood pressure
- Chronic lung disease
- Rheumatic disease
- Diabetes
- Osteoporosis
- High blood lipids/high cholesterol levels
- Cancer
- Mental illnesses
- Inflammatory bowel disease (e.g., Crohn's disease, ulcerative colitis)
- Celiac disease
- No
- Not sure

1. **Do you have one or more of the following diseases?** (You may select more than one option)

- High blood pressure
- Chronic lung disease
- Rheumatic disease
- Diabetes
- Osteoporosis
- High blood lipids/high cholesterol levels
- Cancer
- Mental illnesses
- Inflammatory bowel disease (e.g., Crohn's disease, ulcerative colitis)
- Celiac disease
- No
- Not sure

**Section I. Exposures to Factors Affecting Epithelial Barrier Integrity and Microbiome**

***Eating Habits and Consumed Food Groups***

1. How often do you consume the following foods?

|  | **Once a month**  **or less**  **frequently**  **0 point** | **1–3 times**  **a week**  **-1 point** | **≥4 times a week**  **-2 points** |
| --- | --- | --- | --- |
| Homemade Yogurt |  |  |  |
| Kefir |  |  |  |
| Fresh fruits |  |  |  |
| Fresh vegetables |  |  |  |
| Legumes (e.g. lentils, chickpeas, beans) |  |  |  |
| Unsalted nuts (hazelnuts, walnuts, almonds, peanuts) |  |  |  |
| Homemade pickles |  |  |  |

1. How often do you consume the following packaged or processed products?

|  | **Once a month or less frequently**  **0 point** | **1–3 times**  **a week**  **1 point** | **≥4 times a week**  **2 points** |
| --- | --- | --- | --- |
| Packaged snacks (chips, biscuits, crackers, chocolate, gummies, etc.) |  |  |  |
| Instant meals (ready-made soups, noodles, pizzas, french fries, etc.) |  |  |  |
| Frozen meat products (burgers, chicken nuggets, sausages, etc.) |  |  |  |
| Frozen vegetables (peas, spinach, potatoes, etc.) |  |  |  |
| Pudding mixes, whipped cream, cream cheese, long-life milk |  |  |  |
| Canned foods (beans, fish, tomato paste, etc.) |  |  |  |
| Ready-made sauces (ketchup, mayonnaise, BBQ sauce, etc.) |  |  |  |
| Ready-to-drink beverages (cola, fanta, soda, fruit juice, energy drinks, three in one, two in one coffee mixes, etc.) |  |  |  |

1. How often do you eat takeaway, restaurant, cafe or canteen food?

- Once a month or less frequently **0 point**
- 1–3 times a week  **1 point**
- ≥4 times a week **2 points**

***Cleaning and hygiene habits***

1. Do you think you're very picky or obsessed when it comes to cleanliness?

- Yes **2 points**
- No **0 point**
- Not sure

1. When using toothpaste, do you pay attention to its ingredients (SLS: sodium lauryl sulfate, titanyum dioxide, aspartame or xantham gum, etc.)?

- Yes **0 point**
- No **2 points**

1. How much toothpaste do you use each time?

- The size of a lentil **0 point**
- The size of a hazelnut **1 point**
- That cover the toothbrush **2 points**
- No **0 point**
- I do not know

1. How many times a day do you use toothpaste?

- Once
- Twice
- Three times or more
- I do not know

***Personal Care Habits***

1. Do you use personal care products (e.g., perfume, deodorant, body lotion, etc.)?

- Yes **2 points**
- No **0 points**
- Not sure

1. Do you dye your hair?

- Yes **2 points**
- No (You can skip to question 5) **0 point**

1. How often do you dye your hair?

- Once a month **2 points**
- Once every 3-6 months **1 point**
- Once a year **0 point**

1. How long have you been dyeing your hair?

- Less than 1 year
- 1-5 years
- More than 5 years

1. Do you have a permanent tattoo or makeup?

- Yes **2 points**
- No **0 point** (You can skip to question 7)

1. What is the total diameter (in centimeters) of the tattoo(s) on your body? (You can refer to the image)

- 1-2 cm
- 5 cm
- 10 cm and more


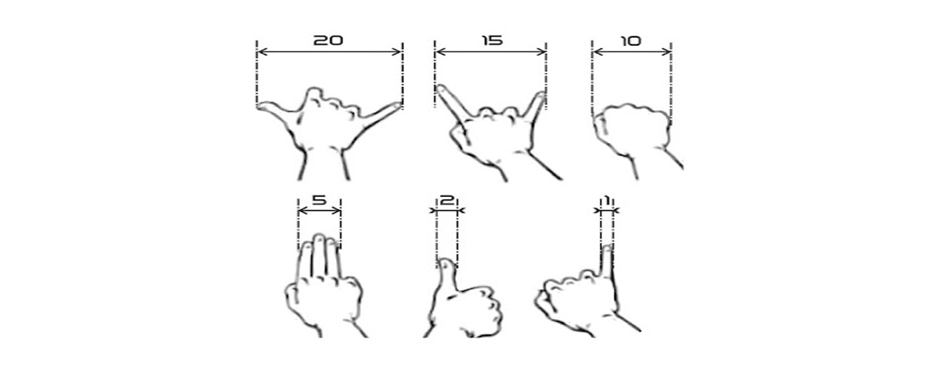


7. Do you wear make-up?

- Yes, heavy make-up **2 points**
- Yes, light make-up **1 point**
- No **0 point**

8. Do you use artificial nails?

- Yes
- No

9. Have you had filler or Botox applied to your skin?

- Yes
- No

***Plastic product usage habits***

**1.**  How often do you store or consume food or drinks in plastic containers?

- Every day **2 points**
- A few times a week **1 point**
- Never/Rarely **0 point**
- I do not know

***Air Pollution Exposure***

1. Which of the following best describes the environment where you live? (Select the most appropriate option)

- In an urban area with heavy car traffic or close to industrial zones **2 points**
- In an urban area with moderate traffic **1 point**
- In a suburban, rural or green area with limited traffic **0 point**
- In a farm/village far from traffic and factories **-1 point**
- Other

1. Where do you usually spend time outdoors? (Choose the most frequent environment)

- In an urban area with heavy car traffic or close to industrial zones **2 points**
- In an urban area with moderate traffic **1 point**
- In a suburban, rural or green area with limited traffic **0 point**
- In a farm/village far from traffic and factories **-1 point**
- Other

1. Do you smoke or use tobacco-related products?

- Yes **2 points**
- No (You can skip to Question 6) **0 point**

1. Which type of tobacco-related products do you use?
   - Cigarettes
   - E-cigarettes
   - Hookah
   - Other
2. How much tobacco-related products do you consume per day?

- Less than 5 **0 points**
- 5-20 **1 point**
- More than 20 **2 points**

1. Are you exposed to secondhand smoke at home?

- Yes **2 points**
- No **0 point**

***Physical activity***

1. How much time do you spend on physical activities in a typical week, including on the way to school, mandatory and voluntary school sports, and other physical activities?

- None **0 point**
- Less than 60 minutes **-1 point**
- 60-120 minutes **-2 points**
- 120-180 minutes **-2 points**
- More than 180 minutes **-2 points**

2. How much time per week do you engage in moderate-intensity physical activities (e.g., activities that make you slightly out of breath)?

- None **0 point**
- Less than 60 minutes **-1 point**
- 60-120 minutes **-2 points**
- 120-180 minutes **-2 points**
- More than 180 minutes **-2 points**

3. How much time per week do you engage in high-intensity physical activities (e.g., activities that make you sweat)?

- None **0 points**
- Less than 60 minutes **-1 point**
- 60-120 minutes **-2 points**
- 120-180 minutes **-2 points**
- More than 180 minutes **-2 points**

**Section II. Awareness about the Epithelial Barrier Theory**

1. Do you read the ingredients of food, cleaning products, or personal care products before buying them?

- Yes
- No (please proceed to question 3)
- Not sure (please proceed to question 3)

1. If yes, what do you usually look for when reading product labels? (You can choose more than one option)

- Expiration dates
- Nutritional content
- Allergen warnings
- Additives / preservatives
- Organic or eco-certified labels
- Approval by a public authority (FDA, EMA, Ministry of Health)
- Recyclable packaging

1. Do you check if a product is BPA-free or made of recyclable material before buying?

- Yes, always
- Sometimes
- No
- I don’t know what BPA is
- I check recycling codes or safety symbols (e.g. triangle with numbers)

1. Do you believe that environmental factors – such as air pollution, packaged food, cleaning or personal care products, or plastic materials – can affect long-term health, such as allergies or chronic diseases?

- Yes
- No
- Not sure

1. Which of the following do you think may negatively affect a child’s long term health if consumed frequently?

- Packaged snacks (biscuits, wafers, candies, chips, etc.)
- Instant meals (soups, noodles, powdered food mixes, etc.)
- Frozen meat products (nuggets, burgers, döner, sausages, etc.)
- Sugary or flavored drinks (sodas, fruit juices, powdered drinks, etc.)
- Ready-made sauces (ketchup, mayonnaise, etc.)
- Ready-to-drink beverages (soda, fruit juice, energy drinks, etc.)
- None of these
- I do not know

1. Have you ever heard of the epithelial barrier theory and its link to allergic or other chronic diseases?

- Yes
- No (thank you for completing the survey)
- Not sure (thank you for completing the survey)

1. Where did you hear about the epithelial barrier theory?

- Doctor or healthcare professional
- Social media / internet
- Television / printed media
- Scientific events / seminars
- School / university
- Friends or family
- Other

1. Have you made any changes in your dietary or cleaning habits based on your knowledge of the epithelial barrier theory?

- Yes
- No
- Sometimes
- I am not sure how to apply it
