# Supplementary material for: A Survey on Environmental Protective and Risk Factors and Awareness Related to Epithelial Barrier Integrity, Microbiome and Allergic Diseases
Source: Allergy. 2025 Dec 23;81(3):930–3. doi: 10.1111/all.70190 (PMC12954555; doi:10.1111/all.70190)
Supplement: Supplementary file 6 — Data S2: Supporting Information. [file ALL-81-930-s002.docx]

**Survey for Parents on Environmental Factors**

**Affecting the Epithelial Barrier Integrity, Microbiome and Allergic Diseases**

Epithelial tissue comprises of cells that line organs such as the intestines, skin, and respiratory tract. Its primary function is to protect our body by forming a barrier against harmful substances like foreign materials and microbes. In recent years, data has been gathered suggesting that the disruption of this barrier contributes to many diseases. Based on your responses to our questions, our goal is to raise awareness on this topic within the community and encourage conscious consumers.

The aim of our study is to assess how much knowledge you have regarding "the protection of the epithelial barrier and its association with diseases," and to learn how much your child is exposed to substances that damage the epithelium.

Participation in this survey is completely voluntary. Your responses will be kept confidential and will only be analysed by the researchers. The information collected from participants will be analysed collectively and used in scientific publications.

I have read the above information and voluntarily agree to participate in this study.

Yes (Thank you for participating in our study)

No

**Survey Instructions:**

Please complete the survey on behalf of your child, whom you have brought to our clinic for an examination. Your identification information will only be used to avoid confusion with other surveys.

**Participant Code Number:**

**General Information**

1. The person completing the survey:

- Mother
- Father

1. Age of the person completing the survey: ………………………….
2. Please select the option that best describes your level of education:

- Literate
- Primary school graduate
- Middle school graduate
- High school graduate
- University graduate
- Postgraduate/Master’s/PhD graduate

1. Please select the option that best describes your monthly income level:

- Less than the minimum wage
- Minimum wage
- 2–3 times the minimum wage
- 4–5 times the minimum wage
- More than 5 times the minimum wage

1. Do you live on a farm with livestock (e.g., cows, sheep, etc.) far from traffic?

- Yes
- No

1. How many people live in your household?

- 1–2
- 3–4
- 5–6
- 7 or more

1. Is there a grandfather or grandmother living with you in your home?

- Yes
- No
- Sometimes

1. What is your child's age range?

- Less than 1 year old
- 1-3 years old
- 4-6 years old
- 7-11 years old
- 12-18 years old

1. What is your child's gender?

- Female
- Male

1. How many siblings does your child have?

- None
- One
- Two
- Three
- Four or more

1. How many of your child's siblings attend school/daycare?

- None
- One
- Two
- Three
- Four or more

1. Do you, your spouse or your other children have any allergic diseases?

- Yes
- No (You can proceed to question 14)
- Not sure (You can proceed to question 14)

1. If yes, which allergic diseases are present? (You may select more than one option)

- Asthma
- Hay fever
- Chronic rhinosinusitis with or without nasal polyps
- Allergic eczema/atopic dermatitis
- Food allergy
- Drug allergy
- Bee allergy
- Urticaria (hives)
- Eosinophilic esophagitis
- Other

1. **Do any of the following diseases exist among the immediate family members? (You may select more than one option)**

- High blood pressure
- Chronic lung disease
- Rheumatic disease
- Diabetes
- Osteoporosis
- High blood lipids/high cholesterol levels
- Cancer
- Mental illnesses
- Inflammatory bowel disease (e.g., Crohn's disease, ulcerative colitis)
- Celiac disease
- No
- Not sure

**Section I. Exposures to Factors Affecting Epithelial Barrier Integrity and Microbiome**

***Perinatal Period***

1. During the pregnancy of the child brought to the allergy clinic, did the mother smoke?

- Yes **2 points**
- No (please proceed to question 3) **0 point**
- I don’t remember (please proceed to question 3)

1. If yes, how many cigarettes did the mother smoke per day during that pregnancy?

- Less than 5 cigarettes per day **0 point**
- 5–20 cigarettes per day **1 point**
- More than 20 cigarettes per day **2 points**
- I don’t remember

1. Did your child’s mother take antibiotics during pregnancy?

- Yes **2 points**
- No **0 point**
- I do not remember

1. What was your child’s mode of delivery?

- Vaginal delivery **-2 points**
- Cesarean delivery **0 point**

1. Was your child hospitalized during the neonatal period (first 28 days)?

- Yes **2 points**
- No (Proceed to Question 7) **0 point**
- I do not remember (Proceed to Question 7)

1. How many days did your child stay in the neonatal ward or NICU?

- Less than 3 days **0 point**
- 3-7 days **1 point**
- More than 7 days **2 points**
- I do not remember

1. Has your child ever received breast milk?

- Yes, exclusively **-2 points**
- Yes, partially **-1 point**
- No (Proceed to Question 9) **0 point**
- I do not remember (Proceed to Question 9)

1. For how long did your child receive breast milk?

- Less than 2 months **0 point**
- 2-6 months **-1 point**
- More than 6 months **-2 points**
- I do not remember

1. Was the child ever given antibiotics during infancy (first 12 months)?

- Yes **2 points**
- No **0 point**
- I do not remember

1. On average, how many times per year has your child used antibiotics so far?

- Once a year or less **0 point**
- 2–5 times a year **1 point**
- More than 6 times a year **2 points**
- I do not remember

1. Did your child start to day care in the first 2 years of his/her life?

- Yes
- No
- I do not remember

1. Do you currently have any cats or dogs in your home?

- Yes **-2 points**
- No **0 point**

***Eating Habits and Consumed Food Groups***

1. How often does your child consume the following foods?

|  | **Once a month**  **or less frequently**  **0 point** | **1–3 times**  **a week**  **-1 point** | **≥4 times a week**  **-2 points** |
| --- | --- | --- | --- |
| Homemade Yogurt |  |  |  |
| Kefir |  |  |  |
| Fresh fruits |  |  |  |
| Fresh vegetables |  |  |  |
| Legumes (e.g. lentils, chickpeas, beans) |  |  |  |
| Unsalted nuts (hazelnuts, walnuts, almonds, peanuts) |  |  |  |
| Homemade pickles |  |  |  |

1. How often does your child consume the following packaged or processed products?

|  | **Once a month or less**  **frequently**  **0 point** | **1–3 times**  **a week**  **1 point** | **≥4 times a week**  **2 points** |
| --- | --- | --- | --- |
| Packaged snacks (biscuits, chocolate, gummies, etc.) |  |  |  |
| Instant meals (ready-made soups, noodles, etc.) |  |  |  |
| Frozen meat products (burgers, chicken nuggets, sausages, etc.) |  |  |  |
| Frozen vegetables (peas, spinach, potatoes, etc.) |  |  |  |
| Baking mixes and ingredients (cake mixes, cream, pudding powders, sauces, food colorings, etc.) |  |  |  |
| Canned foods (beans, fish, tomato paste, etc.) |  |  |  |
| Ready-made sauces (ketchup, mayonnaise, BBQ sauce, etc.) |  |  |  |
| Ready-to-drink beverages (soda, fruit juice, energy drinks, etc.) |  |  |  |

1. What type of oils do you usually use for cooking at home? (You may choose more than one option)

- Solid margarine or vegetable oil (sunflower oil, corn oil, hazelnut oil, canola oil) **2 points**
- Olive oil, butter **0** **point**

1. How often does your child eat takeaway or restaurant food?

- Once a month or less frequently **0 point**
- 1–3 times a week **1 point**
- ≥4 times a week **2 points**

1. At what age did your child first start consuming packaged or processed foods?

- Never
- Before age 1
- Between ages 1-2
- After age 2
- I do not remember

1. Do you use pots, pans, or other utensils for cooking besides steel, cast iron, or glass cookware?

- Yes **2 points**
- No **0 point**

***Cleaning and hygiene habits***

1. Do you consider yourself overly meticulous or obsessive about cleaning at home?

- Yes **2 points**
- No **0 point**
- Not sure

1. How often do you clean your home?

- Every day
- 1-3 times a week
- 1-3 times a month
- I do not know

1. Which types of detergent do you use?

- Powder / Liquid / Tablet / Gel detergent **2 points**
- I do not use any detergent **0 point**
- I do not know

1. Do you use shine liquids in your dishwasher?

- Yes and I do extra rinsing **0 point**
- Yes but I do not do extra rinse **2 points**
- I do not use shine liquid **0 point**
- I do not know

1. What types of laundry detergents or additives do you use? (You may select more than one option)

- Powder / Liquid detergent/Bleach/Fabric softener **2 points**
- Special additive-free detergent **0 point**
- Natural options (e.g., soap granules) **0 point**
- Other
- I do not know

1. Do you use the extra rinse cycle when using the washing machine?

- Yes **-2 points**
- No **0 point**
- I do not know

1. Do you use any cleaning products such as steam cleaning, vinegar, soft soap, or products without detergent for cleaning your home?

- Yes  **0 point**
- **No 2 Points**
- I do not know

1. Do you regularly use shampoo / shower gel/liquid/bar soap/wet wipes/disinfectants, products for sensitive skin or eczema, skin care products (body oil or lotion)/other personal hygiene or cleaning products for your child?

- Yes **2 points**
- No **0 point**
- I do not know

1. Does your child use toothpaste for brushing the teeth?

- Yes **2 points**
- No (Please proceed to **“Plastic product usage habits” part**) **0 point**
- I do not know (Please proceed to **“** **Plastic product usage habits” part**)

1. How many times a day does your child use toothpaste?

- Once
- Twice
- Three times or more
- I do not know

1. How much toothpaste does your child use each time?

- The size of a lentil **0 point**
- The size of a hazelnut **1 point**
- That cover the toothbrush **2 points**
- No **0 point**
- I do not know

***Plastic product usage habits***

**1.**  How often do you store or consume food or drinks in plastic containers?

- Every day **2 points**
- A few times a week **1 point**
- Never/Rarely **0 point**
- I do not know

***Air pollution exposure***

1. Which of the following best describes the environment where your child lives? (Select the most appropriate option)

- In an urban area with heavy car traffic or close to industrial zones **2 points**
- In an urban area with moderate traffic **1 point**
- In a suburban, rural or green area with limited traffic **0 point**
- In a farm/village far from traffic and factories **-1 point**
- Other

1. Where does your child usually spend time outdoors? (Choose the most frequent environment)

- In an urban area with heavy car traffic or close to industrial zones **2 points**
- In an urban area with moderate traffic **1 point**
- In a suburban, rural or green area with limited traffic **0 point**
- In a farm/village far from traffic and factories **-1 point**
- Other

1. Which of the following are regularly present in your home? (You may select more than one)

- Smoking indoors **2 points**
- Use of gas stove, coal or wood stove **2 points**
- Use of biomass (e.g., animal dung, crop residues) for heating **2 points**
- Lack of proper ventilation in the kitchen **1 point**
- None of the above **0 point**

**Section II. Awareness about the Epithelial Barrier Theory**

1. Do you read the ingredients of food, cleaning products, or personal care products before buying them?

- Yes
- No (please proceed to question 3)
- Not sure (please proceed to question 3)

1. If yes, what do you usually look for when reading product labels? (You can choose more than one option)

- Expiration dates
- Nutritional content
- Allergen warnings
- Additives / preservatives
- Organic or eco-certified labels
- Approval by a public authority (FDA, EMA, Ministry of Health)
- Recyclable packaging

1. Do you check if a product is BPA-free or made of recyclable material before buying?

- Yes, always
- Sometimes
- No
- I don’t know what BPA is
- I check recycling codes or safety symbols (e.g. triangle with numbers)

1. Do you believe that environmental factors – such as air pollution, packaged food, cleaning or personal care products, or plastic materials – can affect long-term health, such as allergies or chronic diseases?

- Yes
- No
- Not sure

1. Which of the following do you think may negatively affect a child’s long term health if consumed frequently?

- Packaged snacks (biscuits, wafers, candies, chips, etc.)
- Instant meals (soups, noodles, powdered food mixes, etc.)
- Frozen meat products (nuggets, burgers, döner, sausages, etc.)
- Sugary or flavored drinks (sodas, fruit juices, powdered drinks, etc.)
- Ready-made sauces (ketchup, mayonnaise, etc.)
- Ready-to-drink beverages (soda, fruit juice, energy drinks, etc.)
- None of these
- I do not know

1. Have you ever heard of the epithelial barrier theory and its link to allergic or other chronic diseases?

- Yes
- No (thank you for completing the survey)
- Not sure (thank you for completing the survey)

1. Where did you hear about the epithelial barrier theory?

- Doctor or healthcare professional
- Social media / internet
- Television / printed media
- Scientific events / seminars
- School / university
- Friends or family
- Other

1. Have you made any changes in your dietary or cleaning habits based on your knowledge of the epithelial barrier theory?

- Yes
- No
- Sometimes
- I am not sure how to apply it
